# Genetic control of *Eucalyptus urophylla* and *E. grandis* resistance to canker caused by *Chrysoporthe cubensis*

**DOI:** 10.1590/S1415-47572010005000069

**Published:** 2010-09-01

**Authors:** Lúcio Mauro da Silva Guimarães, Marcos Deon Vilela de Resende, Douglas Lau, Leonardo Novaes Rosse, Alexandre Alonso Alves, Acelino Couto Alfenas

**Affiliations:** 1 Departamento de Fitopatologia/BIOAGRO, Universidade Federal de Viçosa, Viçosa, MGBrazil; 2 Embrapa Florestas, Colombo, PRBrazil; 3 Embrapa Trigo, Passo Fundo, RSBrazil; 4 Veracel S.A., Eunápolis, BABrazil

**Keywords:** genetic resistance, genetic breeding, eucalypt and interspecific hybrids

## Abstract

*Chrysophorte cubensis* induced canker occurs in nearly all tropical and subtropical regions where eucalypts are planted, causing losses in both wood quality and volume productivity, especially so in the warmer and more humid regions of Brazil. The wide inter and intra-specific genetic variability of resistance to canker among *Eucalyptus* species facilitates the selection of resistant plants. In this study, we evaluated resistance to this pathogen in five *Eucalyptus grandis* (G) and 15 *E. urophylla* (U) trees, as well as in 495 individuals from 27 progenies derived from crosses between the trees. In the field, six-months-old test seedlings were inoculated with *C. cubensis*. Lesion length in the xylem and bark was measured eight months later. The results demonstrated that xylem lesions could preferentially be used for the selection of resistant clones. Eight trees (7 U and 1 G) were susceptible, and the remainder (8 U and 4 G) resistant. Individual narrow and broad sense heritability estimates were 17 and 81%, respectively, thereby suggesting that canker resistance is quantitative and highly dependent on dominance and epistasis.

## Introduction

*Chrysophorte cubensis* induced eucalypt canker is one of the most destructive diseases among plantation-raised *Eucalyptus* trees (Van Heerden and Wingfield, 2001). The disease, first reported by Bruner (1917) in Cuba, was initially attributed to *Diaporthe cubensis* Bruner. After the 1970's, occurrence was reported in various regions of the world, but mainly in South America (Hodges *et al.*, 1979; Van der Merwe *et al.*, 2001), Africa (Gibson, 1981; Wingfield *et al.*, 1989; Nakabonge *et al.*, 2006) and southeastern Asia (Sharma *et al.*, 1985; Davison and Coates, 1991). Hodges (1980) proposed transferring the eucalypt canker fungus to *Cryphonectria cubensis* (Bruner) Hodges. However, recent studies involving comparative sequencing of the ITS (Internal Transcribed Spacer) region of ribosomal DNA and β-tubulin genes, revealed this fungus to be phylogenetically distinct from other species of *Cryphonectria* (Myburg *et al.*, 2004)*.* Gryzenhout *et al.*, (2004), on describing the *Chrysoporthe* genera, and so as to accommodate this etiological agent therein, proposed the name *Chrysoporthe cubensis* (Bruner) Gryzenh. & M.J. Wingfield.

The disease is epidemiologically important in regions where the mean temperature is ≥ 23 °C and annual rainfall ≥ 1200 mm (Hodges *et al.*, 1976; Alfenas *et al.*, 1982; Sharma *et al.*, 1985; Conradie *et al.*, 1990). There are three basic symptoms of the canker in eucalypts. The first occurs in plants less than one year old. In this case, the infected plants often die as a consequence of stem girdling and cambium death. The second set of symptoms and signs occurs in trees two years old or more. This set is characterized by the appearance of sunken areas in the stem, cracking of the bark, either at the base of these sunken areas or along the stem, and external colonization of the bark surrounding the dead cambium. The third set of symptoms is the typical canker, a well-defined deep lesion or set of lesions surrounded by calluses. This occurs when a larger section of the cambium is dead, and the tree attempts to recover from the infection (Hodges *et al.*, 1976).

The existence of inter and intra-specific genetic variability for canker resistance in eucalypts (Ferreira *et al.*, 1977; Alfenas *et al.*, 1983; Van Heerden and Wingfield, 2002), together with the development of large scale cloning in the 1980s, has lead to the control of the disease through the selection and cloning of resistant genotypes (Conradie *et al.*, 1990; Wingfield, 1990; Seixas *et al.*, 2004). On the other hand, it has been shown that the combination of favorable environmental conditions and genetic uniformity in clonal plantations may lead to significant losses by this canker, when employing susceptible clones (Van Heerden *et al.*, 2005). Disease monitoring in commercial and experimental plantations, the evaluation of genetic variability in a pathogen population, and the selection of resistant clones and parent trees for breeding programs, are all imperative for reducing potential losses. The aim of this study was to assess the resistance of *E. grandis* and *E. urophylla* parent trees and their progenies.

## Material and Methods

###  Plant material

Five parent trees of *E. grandis* (G39, G45, G58, G93 e G547), 15 of *E. urophylla* (U1177, U1179, U1183, U1185, U1237, U1275, U1282, U1286, U1305, U1310, U1313, U1316, U1392, U1450, U1455), and 495 individuals from 27 progenies derived from crosses between selected parents of these two species, were prepared for resistance testing (Table 1). Ten rooted cuttings from each parent tree and seedlings of the progenies were transplanted to the field and outplanted at a 2.5 x 2.5 m spacing. Test-site location was close to Eunápolis, Bahia, Brazil (≥ 1400 mm annual rainfall and ± 23 °C mean annual temperature). Ten plants from the 367 clone and ten from the 361 *E. grandis* hybrid were used as susceptible and resistant controls, respectively (based on Alfenas, AC – unpublished data). Six months after transplanting, the average breast height circumference was 10.5 cm, large enough for inoculation with *C. cubensis*.

###  Inoculation

A single-spore culture of *C. cubensis* (LPF01), obtained from an *E. grandis* x *E. urophylla* hybrid clone from Belo Oriente, Minas Gerais, Brazil, was used in all inoculations. The fungus was grown in Petri dishes (9 cm in diameter) containing a 2% PDA (Potato-dextrose-agar) medium at 26 ± 1 °C, with a 12-hour photoperiod. Seven millimeters diameter mycelial plugs of this inoculum were then taken from the plates with a cork borer, and inserted into the stem 60 cm above ground-level and just below the bark, in six-month-old test plants (as described in detail by Alfenas *et al.*, 1983). The stem-inoculated area was then enclosed in a humid chamber, consisting of a moistened cotton ball placed below the inoculation point, both covered with plastic film. The plastic film was removed after 30 days.

###  Resistance evaluation

Eight months after inoculation, the plants were decapitated at 1.60 m above ground level, and the length of the bark lesion, caused as a response to inoculation, was measured. Subsequently the stem of each tree was vertically split with a chainsaw and the length of the xylem lesion measured.

###  Statistical analysis

The progenies, parents and control trees were all planted in a completely randomized design. Each experimental unit consisted of a single plant. Resultant data were analyzed with Genes® version 2007.0.0 (Cruz, 2006) and Selegen-Reml/Blup® (Resende, 2002) software packages. Parent trees that did not differ significantly from the resistant control (clone 361) by the Tukey test (p = 0.05) were considered resistant. In the analysis and estimation of genetic parameters, linear mixed models (REML/BLUP procedures, Restricted Maximum Likelihood/Best Linear Unbiased Prediction) were employed. The REML/BLUP adjustment was based on the following mixed model:

y = Xf + Zg + e,

in which *y*, *f*, *g*, *e* are the data, fixed effects (means of control plants and progenies), the genotypic effects in progeny and parent trees (random), and random error vectors, respectively, whereas *X* and *Y* are the incidence matrixes for *f* and *g*, respectively. Fitting this model to the experiment, with both progeny and parent trees, enabled estimating variance components (by REML) and broad and narrow sense heritabilities.

The following variance structures and relations were obtained through separate analysis of the full-sib experiment:

(i) Genetic variance among full-sib families: 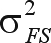.

(ii) Full-sib family mean heritability: 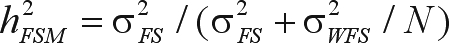, where 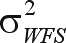 is within family individual phenotypic variation and *N* is the number of plants per family.

(iii) Accuracy of family selection: 
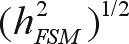
.

(iv) Coefficient of genotypic variation among progenies: 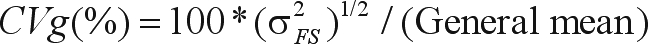.

(v) Coefficient of residual variation: 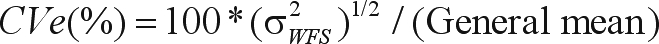.

(vi) Within full-sib family individual broad sense heritability: 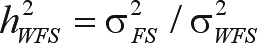, assuming that between and within family genetic variances are approximately the same.

Using a separate analysis for the cloned parents experiment, the following variance structures and relations were obtained:

(vii) Genetic variance among cloned parents: 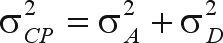.

(viii) Individual broad sense heritability: 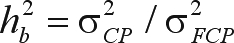, where 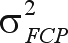 is the individual phenotypic variation for parents.

Furthermore, joint analysis of both experiments (cloned parents and full-sib families) revealed the possibility of estimating additive genetic variance ( 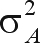) by its isolation from the sum of both itself and dominance variance. The three types of covariance between relatives (full-sibs, cloned parents and parent-offspring) were used simultaneously for estimating { 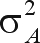} by using residual maximum likelihood (REML). Specifically, the following estimates were obtained:

(ix) Additive genetic variance from joint analysis: 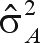.

(x) Dominance genetic variance also from joint analysis: 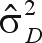.

(xi) Narrow-sense individual heritability - 
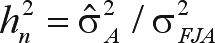
, where 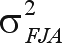 is individual phenotypic variation from joint analysis.

(xii) Broad-sense individual heritability from joint analysis: 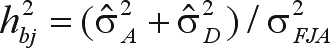.

The effects of segregation resulting from crossing highly heterozygous individuals were estimated by the difference between the mean of clonally analyzed parent trees and the mean of each cross involving the very same parent trees. These effects provide estimates of the depression by segregation resulting in the reduction in character average. This can be understood as the loss of heterosis, which may occur when crossing individuals that are predominantly heterozygotes. The genotypic mean values of the parent trees (assessed clonally) used in calculations also include the effects of dominance, and not only the additive effects that would be expected if parent trees were assessed seminally, as is common in annual crop breeding.

## Results

**Figure 1:**
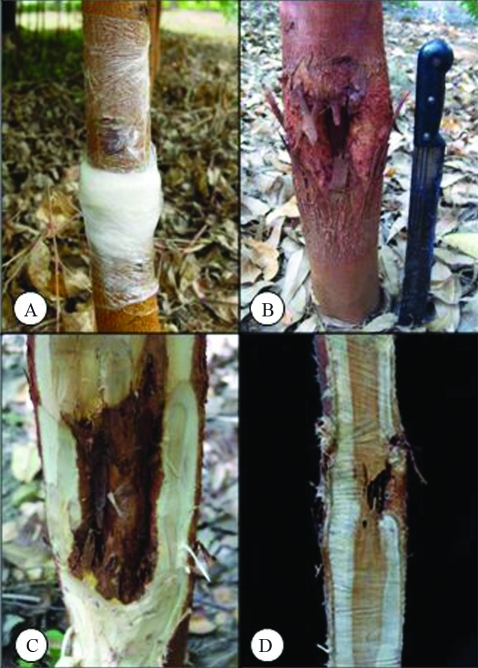
(A) Moist chamber, (B) typical canker, (C) bark lesion, and (D) xylem lesion.

Bark and sapwood symptoms were typical of the disease through natural infection (Figure 1). The colonization of *C. cubensis* in host tissues was confirmed by re-isolating the fungus on PDA. The correlation between the length of bark and xylem lesions in parent trees was virtually nil (0.09), whereas in progeny this was 0.68, with a similar trend for average length (Table 1).

**Figure 2:**
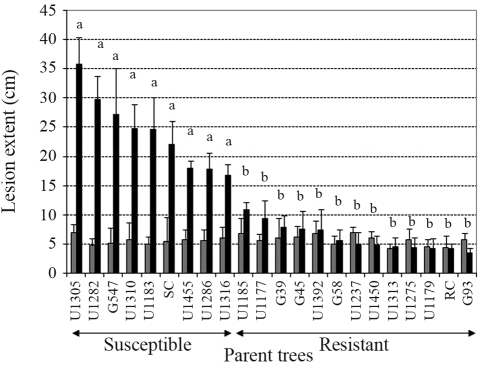
Mean and standard deviation for extension of bark (gray) and xylem (black) lesions in parent trees of *Eucalyptus grandis* (G) and *E. urophylla* (U) inoculated with *Chrysoporthe cubensis*. SC – susceptible control and RC – resistant control. Parent trees followed by the same letter did not differ by Tukey test (p = 0.05). Parent trees were considered resistant when they did not differ statistically from resistant control.

G93 was the most resistant among the parent trees assessed, with a mean xylem lesion length inferior to that of resistant control (RC) (Figure 2). Besides G93, the parent trees U1179, U1275, U1313, U1450, U1237, G58, U1392, G45, G39, U1177 and U1185 were considered resistant, since lesion lengths did not differ statistically from those of RC. The remaining parent trees (U1316, U1286, U1455, U1183, U1310, G547, U1282 and U1305) were susceptible, and in the case of U1183, U1310, G547, U1282 and U1305, mean xylem lesion lengths even exceeding those of susceptible control (SC) (Figure 2). All progenies segregated for resistance; however, none of them had a mean xylem lesion size below the mean of the resistant control (4.7 cm) (Table 1). Furthermore, in ten progenies (P13, P12, P01, P06, P14, P22, P23, P19, P25 and P27) lesions were larger than in susceptible control (22 cm) (Table 1). In the six progenies (P09, P20, P11, P18, P05 and P08) from crosses between the resistant parent trees, mean xylem lesion sizes were the lowest (Table 1).

The estimates of individual heritability in a narrow (inter-specific level) and broad (intra-specific level) sense were 17% and 80%, respectively (Table 2). There was a large genotypic variation among families (genotypic variation coefficient equal to 39%), thereby indicating high heritability (65%) and accuracy (81%) for selection among families. There is also some genetic variability for selection within families, as corroborated by the estimate (8%) for heritability (Table 3). Estimates of heterosis loss or segregation effect in crosses between highly resistant heterozygous individuals were 78% on an average (Table 3).

## Discussion

In view of the correlations encountered between the extent of lesions in bark and xylem alike (in the evaluated progenies), the method of inoculation used in this study proved to be appropriate for detecting resistance variability in the *Eucalyptus* spp. x *C. cubensis* pathosystem. In xylem, these lesions were more extensive, thus constituting the preferential criterion when selecting resistant clones. Nevertheless, heritability of lesion extent in bark in both experiments (parent trees and progenies) tended to zero, thereby inferring that this variable is inappropriate for genotypic discrimination.

This study revealed wide genetic variation for resistance in *E. grandis* and *E. urophylla*, thereby corroborating previous results (Ferreira *et al.*, 1977; Alfenas *et al.*, 1983; Van Heerden and Wingfield, 2002). Twelve out of the twenty parent trees tested were resistant. This high number of resistant parents probably reflects the intense selection for resistance to this disease that has taken place over the last few years (Van Zyl and Wingfield, 1999). It is noteworthy that these parents possess not only favorable alleles for resistance, since there was segregation in their progenies, but also that this trait can be transmitted through crossing with other resistant parents.

When assessing the nature and magnitude of those gene effects controlling a specific character, it is important to select and predict the behavior of hybrid generations and segregating populations. An estimation of the proportion of variability attributed to additive, dominant and epistatic effects is crucial, since the relative importance of these factors exerts a strong influence on genetic breeding programs. However, although the eucalypt canker is a disease of recognized importance, there are only a few studies aiming to obtain genetic information for resistance to this disease. In the present case, estimates of individual heritability in the narrow (inter-specific level) and broad (intra-specific level) sense were equivalent to 17% and 81%, respectively, thus allowing the following inferences: (i) due to the low level of additive heritability and high level of broad sense heritability, breeding for eucalypt canker resistance may be achieved mainly by the selection and cloning of highly resistant genotypes, (ii) this resistance is probably a multigenic character; and (iii) this character exhibits high allelic dominance, or epistasis, given the wide distance between the values of the two heritabilities.

Additive genetic determination of resistance was 17%, and of dominance and epistasis 64%. Borges and Brune (1981), when using data from natural infection, also studied the heritability of resistance to eucalypt canker in half-sib families of *E. grandis*. However, according to these authors, heritability was of reasonable magnitude (0.65), with values in the narrow and broad sense close to one another. The results here are quite distinct from those of Borges and Brune (1981), indicating that genetic control of the character may vary between different sources of resistance. These contrasts may also be partially attributed to disease escape, since Borges and Brune (1981) only evaluated naturally infected plants.

These issues should be carefully considered in breeding programs, for the more efficient selection of resistant clones. We found that the mean genotypic values of progeny as regards to xylem canker length exceeded those of parents, thereby indicating an increase in susceptibility. The loss of heterosis effect (78%) may be explained by the presence of allelic dominance towards greater resistance to canker. Thus, crosses between superior heterozygous genotypes cause, on an average, a decrease of 50% in the total contribution of heterozygous loci to the character, possibly explaining the observed depression by segregation. Another possibility, and which may occur simultaneously, is the ‘break', through hybridization, of favorable epistatic combinations for resistance in pure species. These epistatic combinations involve favorable polygenic blocks established during the long evolutionary process of the species and result in a co-evolution of genes. In temperate climates, inter-specific hybridization of eucalypt species has resulted in greater susceptibility to diseases in hybrids (Borralho, 2007).

Due to the wide genetic variation for canker resistance in *E. grandis* and *E. urophylla*, the introduction of resistant parent trees into ongoing breeding programs may increase the chances of obtaining disease resistant clones at the end of the selection program. The results of our study also reinforce the need for using artificial inoculation with *C. cubensis*, when selecting pathogen-resistant parent trees and progenies of *Eucalyptus* spp. Furthermore, they also highlight the importance of cloning resistant genotypes for disease control. Although we used only one isolate of *C. cubensis* in this study, others have disclosed diversity in the pathogen population (Van Zyl *et al.*, 1998) and the existence of specific pathogen x host interactions, resulting in differential interactions between eucalypts clones and *C. cubensis* isolates (Alfenas *et al.*, 1983; Van Heerden *et al.*, 2005). Therefore, isolates with a broader virulence spectrum should be used in future assays for identifying resistant eucalypt clones.

## Figures and Tables

**Table 1:** Extent of xylem lesion in hybrid families of *Eucalyptus grandis* x *E. urophylla* inoculated with *Chrysoporthe cubensis*.

Progeny	Parent tree (resistance phenotype)	N° of plants evaluated per progeny	Mean (cm)	Lesion size		Stand. deviation
				Maximum (cm)	Minimum (cm)	
	RC	10	4.7	22	2	5.4
P09	G39 (R) x U1450 (R)	20	9.9	29	2	8.7
P20	U1237 (R) x G93 (R)	20	10.0	48	2	12.8
P11	G45 (R) x U1450 (R)	19	11.6	60	2	17.9
P18	U1185 (R) x G83 (-)	20	11.8	37	2	11.0
P05	G39 (R) x U1275 (R)	14	13.1	60	2	16.6
P08	G39 (R) x U1313 (R)	21	13.6	55	2	17.6
P26	U1310 (S) x G547 (S)	21	14.3	60	2	16.6
P16	U1185 (R) x G47 (-)	19	14.5	60	2	13.4
P24	U1310 (S) x G58 (R)	17	14.8	60	2	15.7
P15	U1179 (R) x G549 (-)	18	15.8	40	2	12.0
P21	U1286 (S) x G99 (-)	19	17.3	60	2	16.5
P07	G39 (R) x U1305 (S)	16	17.4	60	2	19.2
P03	G39 (R) x U1183 (S)	21	17.6	60	2	15.9
P17	U1185 (R) x G51 (-)	19	18.4	60	2	17.7
P02	G39 (R) x U1072 (-)	20	18.6	62	2	15.7
P10	G45 (R) x U1177 (R)	19	20.4	63	2	21.3
P04	G39 (R) x U1206 (-)	19	21.9	54	2	20.0
	SC	10	22.0	43	2	13.2
P27	U1412 (-) x G549 (-)	19	22.1	67	2	20.1
P25	U1310 (S) x G93 (R)	19	22.8	64	2	24.8
P19	U1185 (R) x G99 (-)	18	23.4	94	2	24.2
P23	U1310 (S) x G51 (-)	17	24.1	86	2	28.2
P22	U1286 (S) x G504 (-)	19	24.7	55	2	17.1
P14	U1179 (R) x G547 (S)	9	26.1	60	2	22.7
P06	G39 (R) x U1282 (S)	20	26.4	60	2	17.9
P01	G39 (R) x U1034 (-)	19	28.1	60	2	22.7
P12	G47 (-) x U1455 (S)	20	30.8	61	4	18.7
P13	G99 (-) x U1316 (S)	13	40.1	60	4	22.3

(R) Resistant parent tree and (S) susceptible parent tree, based on a Tukey test (p= 0.05) (Figure 1); and (-) parent tree resistance not evaluated. (G) *E. grandis*; (U) *E. urophylla*; (RC) resistant control and (SC) susceptible control.

**Table 2:** Estimates of genetic parameters (variance components, heritabilities and coefficients of genetic variation) of *E. grandis x E. urophylla* families for canker (*Chrysoporthe cubensis*) resistance, assessed by measuring xylem lesion length (cm).

Genetic parameters	Values
Genotypic variance among full-sib progenies	30.40
Genotypic variance among cloned parent trees	45.29
Individual phenotypic variance for progenies	360.10
Individual phenotypic variance for parent trees	55.88
Individual narrow sense heritability	0.17 ± 0.07
Individual broad sense heritability	0.80 ± 0.25
Individual phenotypic variance within progenies	329.70
Individual heritability within progenies	0.08
Heritability of progeny means	0.65
Accuracy of progeny means	0.81
Genotypic variation coefficient among progenies (%)	39.38
Residual variation coefficient (%)	29.00
Relative variation coefficient (%)	1.36

**Table 3:** Genotypic values (GV) of families and parent trees of *Eucalyptus grandis* (G) and *E. urophylla* (U), and values for segregation effects (loss of heterosis for resistance) of crosses involving two parents that were evaluated for resistance to eucalypt canker caused by *Chrysoporthe cubensis* by measuring the extent of xylem lesion (cm).

Female parent	Male parent	Family GV	Female parent GV	Male parent GV	Aver. GV of the PT*	Loss of heterosis	Loss of heterosis (%)
G39	U1450	12.91**	7.97	6.64	7.30	-5.61	-76.81
U1237	G93	12.94	6.64	6.02	6.33	-6.62	-104.56
G45	U1450	14.14	7.88	6.64	7.26	-6.88	-94.83
G39	U1313	15.38	7.97	6.55	7.26	-8.12	-111.95
G39	U1275	15.58	7.97	6.46	7.21	-8.37	-116.02
U1310	G547	15.87	15.49	16.55	16.02	0.15	0.92
U1310	G58	16.39	15.49	6.99	11.24	-5.15	-45.77
G39	U1183	18.11	7.97	22.52	15.24	-2.87	-18.85
G39	U1305	18.14	7.97	20.36	14.16	-3.97	-28.05
G45	U1177	20.06	7.88	8.67	8.28	-11.78	-142.4
U1310	G93	21.65	15.49	6.02	10.75	-10.89	-101.27
U1179	G547	22.65	6.37	16.55	11.46	-11.19	-97.59
G39	U1282	24.14	7.97	17.71	12.84	-11.31	-88.09
Mean	17.54	9.47	11.36	10.41	-7.12	-78.49	

*Average genotypic value of parent trees. **Higher values indicate higher susceptibility to the disease.
